# Supplementary material for: Ablation-resistant carbide Zr0.8Ti0.2C0.74B0.26 for oxidizing environments up to 3,000 °C
Source: Nat Commun. 2017 Jun 14;8:15836. doi: 10.1038/ncomms15836 (PMC5474735; doi:10.1038/ncomms15836)
Supplement: Supplementary Information — Supplementary Figures, Supplementary Notes and Supplementary References [file ncomms15836-s1.pdf]

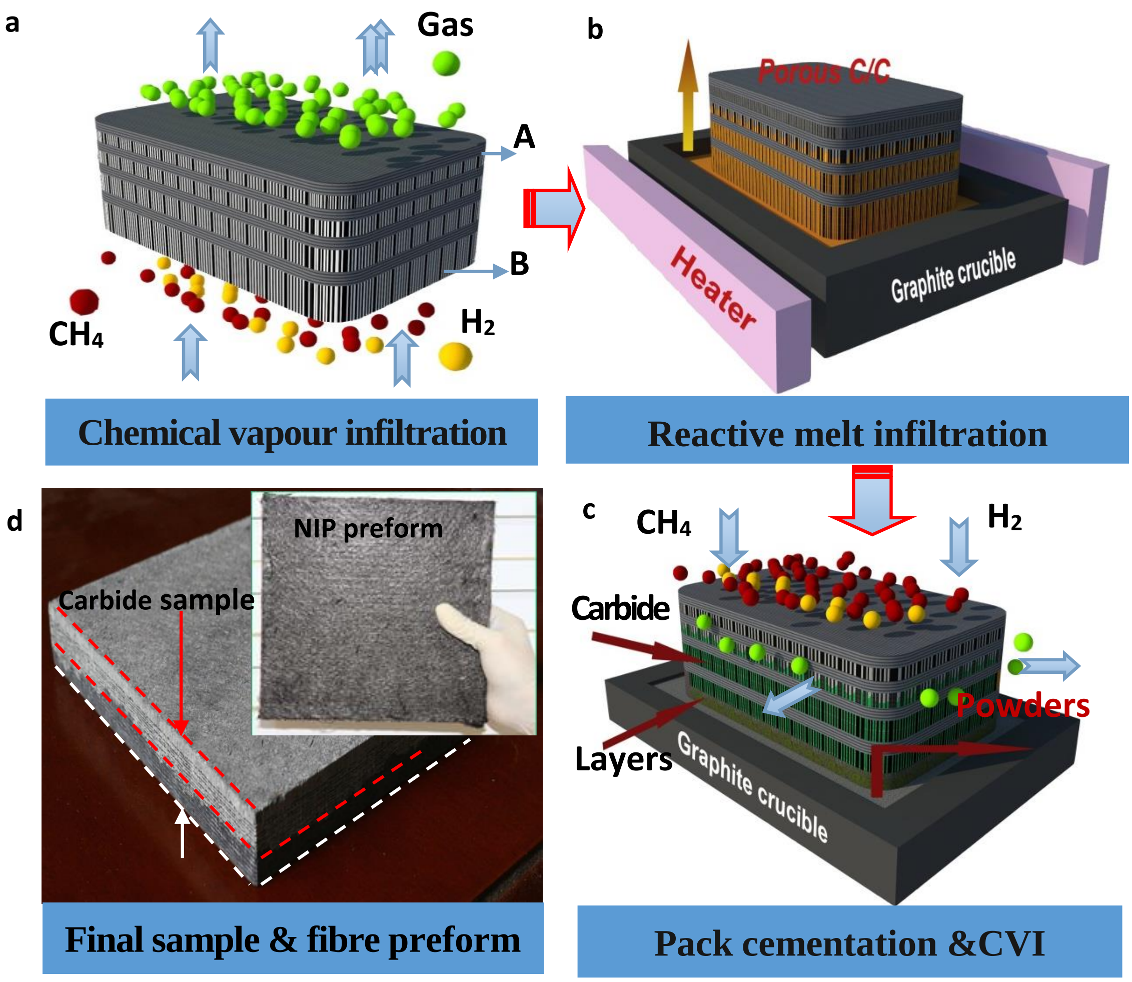

Supplementary Figure 1. Schematic representation of the sample preparation process. (a) A carbon fibre preform was densified by chemical vapour infiltration (CVI). (b) The preform was reaction melt infiltrated to create a C/C-carbide composite. (c) Onto this a carbide layer was formed by pack cementation and the carbon-based part further densified by CVI. (d) Photographs of the carbon fibre preform and the final test-piece with the carbide layer uppermost (grey area) and bottom of C/C composite (black area).

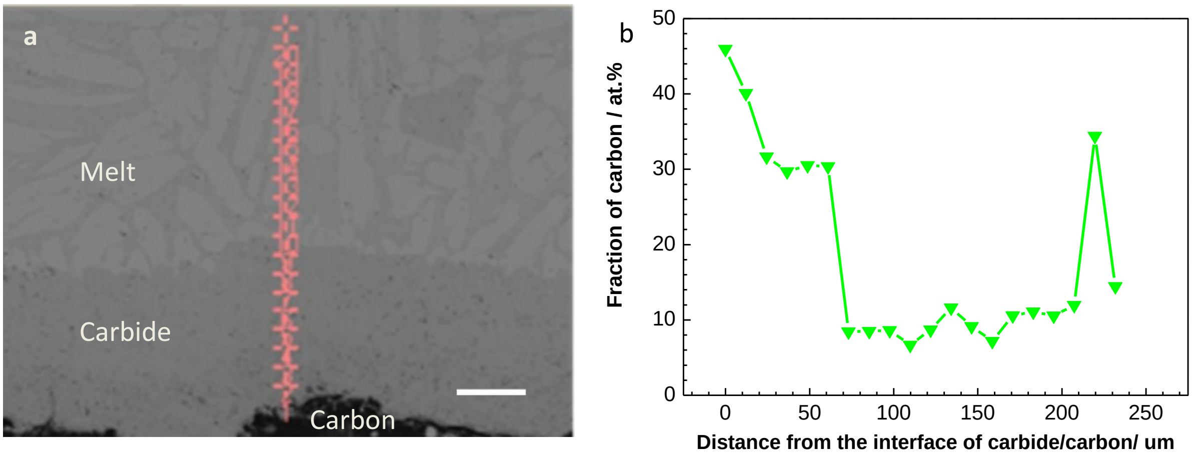

Supplementary Figure 2. Distribution of carbon concentration of $Zr_{0.8}Ti_{0.2}C_{(1-x)}$ in the interface between solid melt and carbide. (a) Interface between melt and carbide. (b) Distribution of carbon concentration in $Zr_{0.8}Ti_{0.2}C_x$. The value from about 48.5% at the interface between carbon and carbide sharply decreases to about 32.0% at the interface between carbide and melt. The distribution of carbon concentration in (a), (b), together with the Zr-C [1] and Ti-C [2] phase diagrams showing in MSI Eureka database in SpringerMaterials, indicate non-stoichiometric carbide tends to form when Zr-Ti melts reacts with carbon. Scale bar, 50 μm.

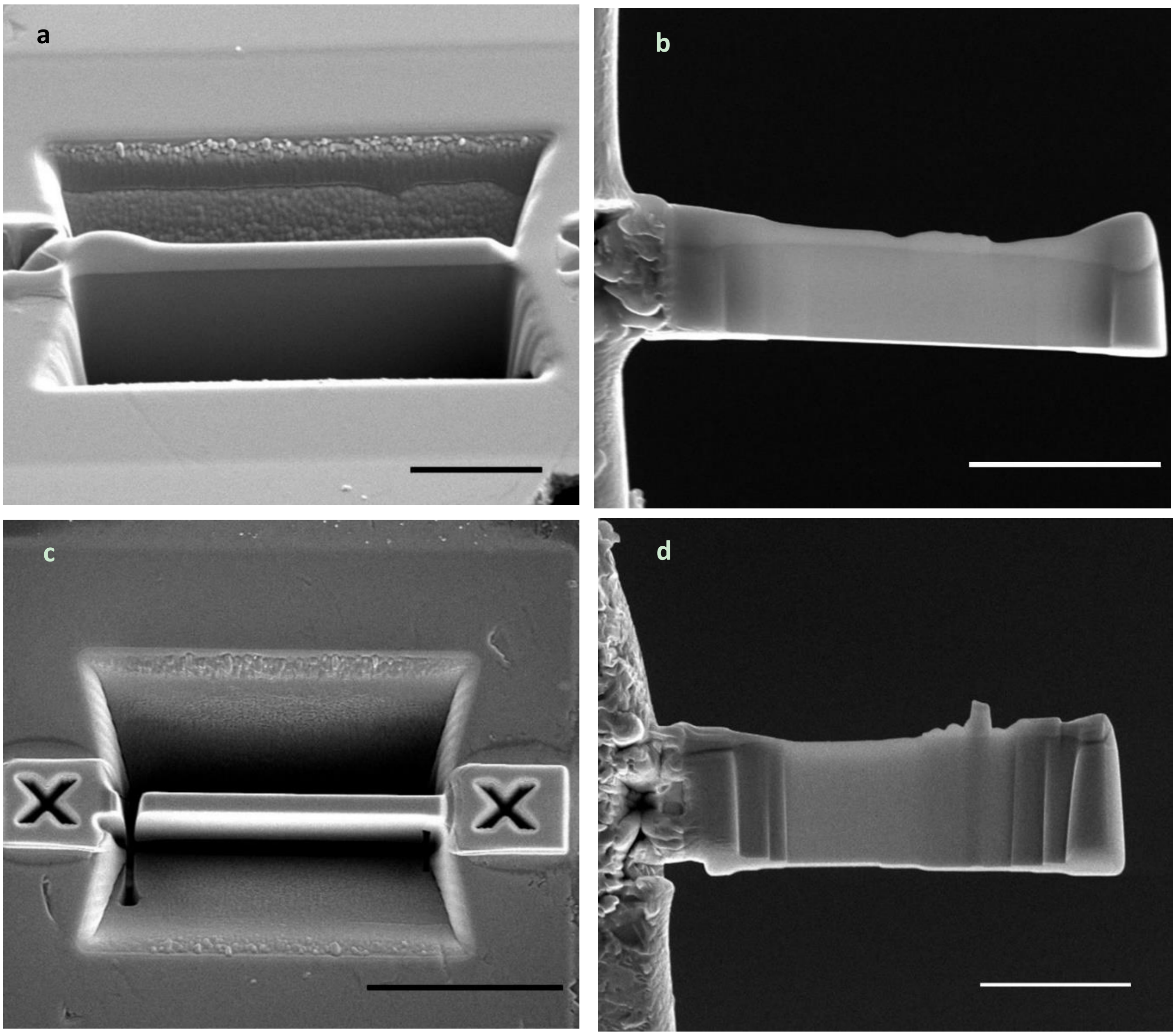

Supplementary Figure 3. FIB images of $Zr_{0.8}Ti_{0.2}C_{(1-x)}$ and $Zr_{0.8}Ti_{0.2}C_{0.74}B_{0.26}$ sample. (a) and (b) $Zr_{0.8}Ti_{0.2}C_{(1-x)}$. (c) and (d) $Zr_{0.8}Ti_{0.2}C_{0.74}B_{0.26}$ sample. Scale bar in a,b,d, 5 μm. Scale bar in c, 10 μm.

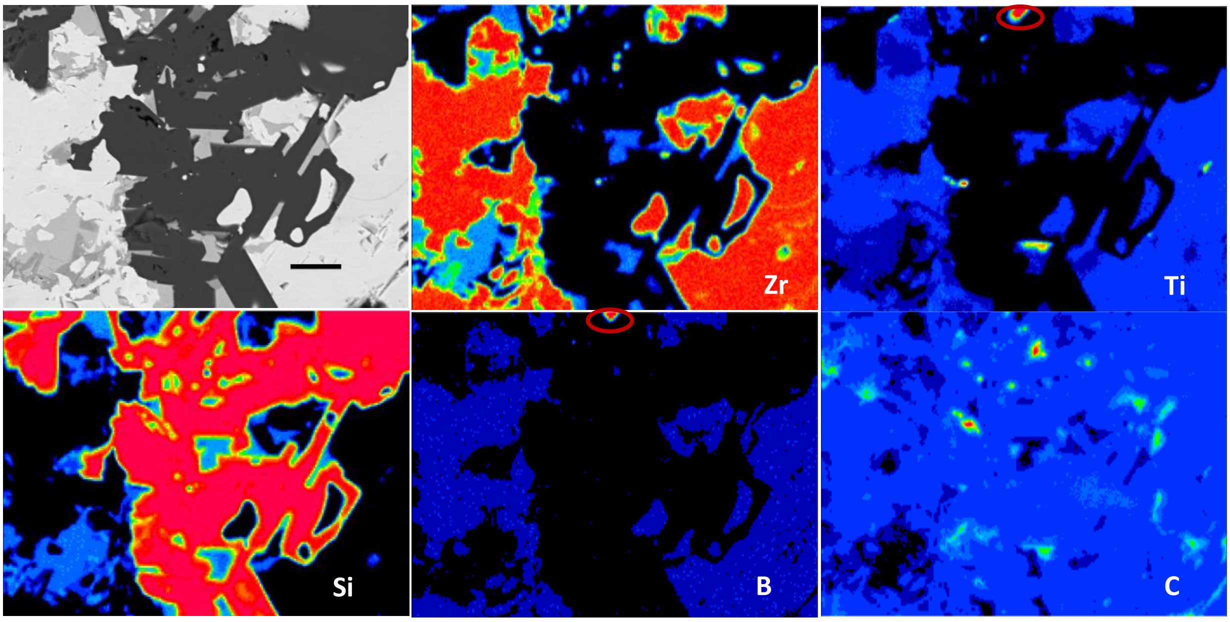

Supplementary Figure 4. SEM microstructure of $Zr_{0.8}Ti_{0.2}C_{0.74}B_{0.26}$/SiC ceramic with colour maps showing various elemental maps. In red ovals, the atom ratio of Ti to B is about 0.5 (TiB_2), according to spot detected by EPMA. Scale bar, 10 μm.

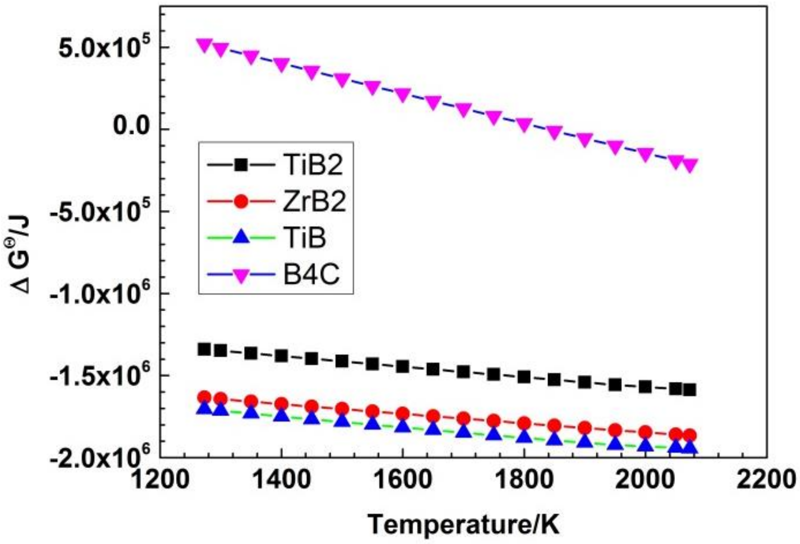

Supplementary Figure 5. Gibbs energy of formation of boride and carbide, according to the reactions of (1), (2) and (3) in main text, indicating that at over 1800 K, all the reactions could happen.

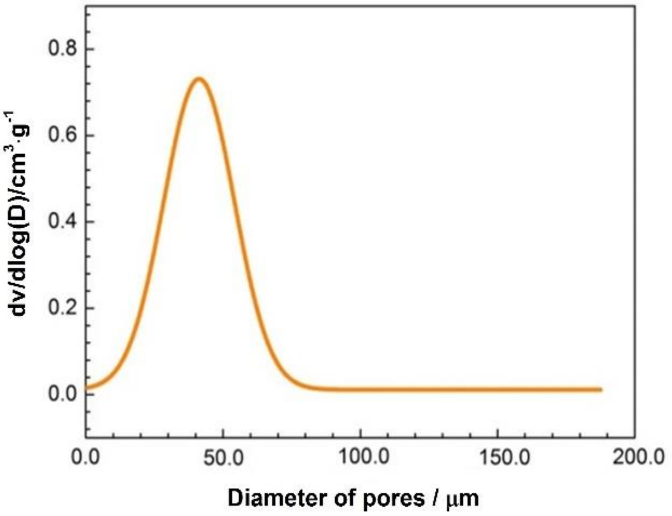

Supplementary Figure 6. Size distribution of open pores in composite with NIP having a density of 1.16 g cm^{-3}.

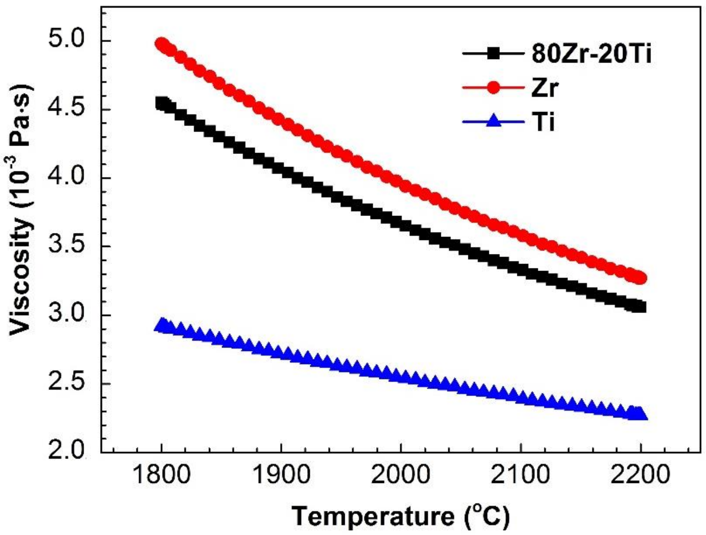

Supplementary Figure 7. Viscosity of Zr, Ti and 80 at. % Zr –20 at. % Ti melts as function of temperature. The viscosities of Zr and Ti were measured by Ishikawa et al [3], and the viscosity of binary alloy melt of Zr-Ti was calculated according to Moelwyn-Hughes and Iida methods [4]. More details about the calculation can be found in Ref. 5.

Supplementary Notes

Supplementary Note 1: Preparation of other materials for ablation property comparison

C/C composites with needled integrated preform were fabricated using CVI process with CH_4 and H_2 at 900–1,000 ℃. C/C-SiC composites were prepared using the RMI process at the temperature of 1,500-1,800 ℃. Firstly, the bulk carbon preforms were densified by pyrocarbon to porous C/C composites with a density of 1.0-1.3 g cm^{-3} using CVI process, and then the SiC were introduced into the C/C composites by RMI process in argon at 1,500–1,800 ℃. Similarly, C/C-$Zr_{0.8}Ti_{0.2}C$ composites were approached by the CVI and RMI process using the Zr 80 at. % -Ti 20 at. % powder mixture at the temperature of 1,900-2,100 ℃. The powders and porous C/C composites were heated inside a graphite crucible by an electric furnace. During RMI, the Zr-Ti melts and Zr-Ti-Si were infiltrated into the C/C composites and reacted with the pyrocarbon to form the $Zr_{0.8}Ti_{0.2}C$ and ZrTiC-SiC, respectively. ZrB_2-SiC ceramics were prepared using the SPS. The starting powders were ZrB_2 powder with a purity of > 99.5% and average particle size of 15 μm, and SiC powder with a purity of > 99% and average particle size of 0.55 μm. 20 vol% SiC combined with ZrB_2 powder were ball-milled. After mixing, the slurry was dried by vacuum oven. The obtained powder mixtures were loaded into graphite die, then spark plasma sintered. The samples were heated from room temperature to 1,580 ℃ and held for 2 min at a uniaxial pressure of 25MPa [6].

Supplementary Note 2: Formation mechanisms of SiC during PC process

$$Si_{(s,l,g)} + C_{(s)} \longrightarrow SiC_{(s)} \quad \text{(S1)}$$

$$Al_2O_{3(s)} + 2Si_{(l)} \longrightarrow 2SiO_{(g)} + Al_2O_{(g)} \quad \text{(S2)}$$

$$SiO_{(g)} + C_{(s)} \longrightarrow SiC_{(s)} + CO_{(g)} \quad \text{(S3)}$$

During the PC process, silicon solids, gas and melts would react with pyrocarbon and carbon fibres. Additionally, the doping agent such as Al_2O_3 would react with silicon and form the SiO gas to promote the reaction in Equation S3.

Supplementary References

1. Duschanek, H., Rogl, P. & MSIT®. System name: C-Zr. SpringerMaterials.
2. Lukas H.L. & MSIT®. System name: C-Ti. SpringerMaterials.
3. Ishikawa, T., Paradis P.F., Okada J.T. & Watanabe Y. Viscosity measurements of molten refractory metals using an electrostatic levitator. Meas. Sci. Technol. 23, 1-9 (2012).
4. Roderick I. L. & Takamichi IIDA. The physical properties of liquid metals, Oxford University Press, 1988.
5. Zeng Y. et al. Preparation and microstructure of carbon/carbon composites modified with Zr-Ti-C fabricated by reacted melt infiltration. The 8th Pacific Rim international congress on advanced materials and processing, Hawaii, USA, 447-456(2013).
6. Zhang X, Chen Z.K, Xiong X., Liu R.T. & Zhu Y.Y. Morphology and microstructure of ZrB_2–SiC ceramics after ablation at 3000 ℃ by oxy-acetylene torch. Ceram. Intern. 42(2), 2798-2805 (2016).
